# Apoyo social percibido por mujeres gestantes de Santa Marta, Colombia: un análisis comparativo.

**DOI:** 10.15649/cuidarte.2448

**Published:** 2023-03-29

**Authors:** Whitne Dayana Thomas-Hilarión, Laura Vanessa Fuentes-Vanegas, Yeison David Gallo-Barrera, Ediltrudis Ramos De la Cruz

**Affiliations:** 1 . Universidad del Magdalena, Santa Marta, Colombia. E-mail: wdthomashilarion@gmail.com Universidad del Magdalena Universidad del Magdalena Santa Marta Colombia wdthomashilarion@gmail.com; 2 . Universidad del Magdalena, Santa Marta, Colombia. E-mail: laurafuentesvanegas@gmail.com Universidad del Magdalena Universidad del Magdalena Santa Marta Colombia laurafuentesvanegas@gmail.com; 3 . Universidad del Magdalena, Santa Marta, Colombia. E-mail: yeisondgb@gmail.com Universidad del Magdalena Universidad del Magdalena Santa Marta Colombia yeisondgb@gmail.com; 4 . Universidad del Magdalena, Santa Marta, Colombia. E-mail: eramos@unimagdalena.edu.co Universidad del Magdalena Universidad del Magdalena Santa Marta Colombia eramos@unimagdalena.edu.co

**Keywords:** Trimestres del Embarazo, Apoyo Social, Afecto, Estudio Comparativo, Colombia., Pregnancy Trimesters, Social Support, Affect, Comparative Study, Colombia., Trimestres da Gravidez, Apoio Social, Afeto, Estudo Comparativo, Colómbia.

## Abstract

**Introducción:**

El apoyo social percibido durante la gestación es importante para la salud mental perinatal. Sin embargo, poco se conoce sobre estas variables en la población colombiana.

**Objetivo:**

Comparar el apoyo social percibido según variables sociodemográficas, ginecobstétricas y afecto positivo en mujeres gestantes de Santa Marta, Colombia.

**Materiales y métodos:**

Participaron 40 mujeres entre 19 y 41 años (M=26.48; DE=5.03), que se encontraban en su tercer trimestre de embarazo, beneficiarias de un programa de promoción de la lactancia materna exclusiva. Las participantes diligenciaron una ficha de información sociodemográfica y ginecobstétrica, la Escala Multidimensional de Apoyo Social Percibido (a=0.88) y una subescala de la Escala de Afectos Positivos y Negativos (a=0.82). Se aplicó la prueba U de Mann-Whitney para los análisis estadísticos comparativos y se siguieron los lineamientos éticos en investigación con humanos.

**Resultados:**

67% de las participantes fueron de estrato socioeconómico bajo, 87.5% tenía pareja, 67.5% contaba con estudios superiores, 42.5% tenía trabajo y 47.5% eran madres primerizas. Las mujeres de estrato socioeconómico alto percibieron mayor apoyo social por parte de sus amigos (p=0.01). El apoyo social familiar fue significativamente más alto en las madres primerizas (p=0.01) y en las que reportaron mayor afecto positivo (p=0.03). Por último, el apoyo social por parte de personas significativas fue mayor cuando era el primer embarazo de la mujer (p=0.02).

**Conclusiones:**

Se encontraron diferencias significativas en algunas dimensiones del apoyo social percibido según el estrato socioeconómico, ser madre primeriza y el afecto positivo. Se recomienda realizar otros estudios con mayor tamaño muestral.

## Abstract (en)

**Introduction:**

Perceived social support during gestation is important for perinatal mental health. However, little is known about these variables in the Colombian population.

**Objective:**

To compare perceived social support with sociodemographic and gynaeco-obstetric variables and positive affect in pregnant women in Santa Marta, Colombia.

**Materials and Methods:**

Forty women between 19 and 41 years of age (M=26.48; SD=5.03), in their third trimester of pregnancy and beneficiaries of a program to promote exclusive breastfeeding, participated. The participants filled out a sociodemographic and gynaeco-obstetric form, the Multidimensional Scale of Perceived Social Support (MSPSS) (a=0.88), and a subscale of the Positive and Negative Affect Schedule (PANAS) (a=0.82). The Mann-Whitney U test was used for comparative statistical analyses. Ethical guidelines for human research were followed.

**Results:**

67% of the participants belonged to a low socioeconomic status, 87.5% had a partner, 67.5% had a higher education, 42.5% were employed, and 47.5% were new mothers. Women of higher socioeconomic status perceived greater social support from their friends (p = 0.01). Family social support was significantly higher in first-time mothers (p=0.01) and mothers who reported higher positive affect (p = 0.03). Finally, social support from significant others was higher when it was the woman's first pregnancy (p=0.02).

**Conclusions:**

Significant differences were found in some dimensions of perceived social support according to socioeconomic status, being a first-time mother, and positive affect. Further studies with larger sample sizes are recommended.

## Resumo

**Introdujo:**

O apoio social percebido durante a gravidez é importante para a saúde mental perinatal. No entanto, pouco se sabe sobre essas variáveis na populagáo colombiana.

**Objetivo:**

Comparar o apoio social percebido segundo variáveis sociodemográficas, ginecológicas e de afeto positivo em gestantes de Santa Marta, Colombia.

**Materiais e métodos:**

Participaram 40 mulheres entre 19 e 41 anos (M=26,48; DP=5,03), que estavam no terceiro trimestre de gestagáo, beneficiárias de um programa de promogáo do aleitamento materno exclusivo. As participantes preencheram uma ficha sociodemográfica e ginecológica, a Escala Multidimensional de Suporte Social Percebido (a=0,88) e uma subescala da Escala de Afetos Positivos e Negativos (a=0,82). O teste U de Mann-Whitney foi aplicado para análise estatística comparativa e foram seguidas as diretrizes éticas para pesquisa em humanos.

**Resultados:**

67% das participantes eram de baixo nível socioeconomico, 87,5% tinham companheiro, 67,5% tinham ensino superior, 42,5% trabalhavam e 47,5% eram máes de primeira viagem. As mulheres de nível socioeconomico alto perceberam maior apoio social dos amigos (p=0,01). O apoio social familiar foi significativamente maior nas novas máes (p=0,01) e naquelas que relataram maior afeto positivo (p=0,03). Por fim, o apoio social de pessoas significativas foi maior quando se tratava da primeira gravidez da mulher (p=0,02).

**Conclusóes:**

Foram encontradas diferengas significativas em algumas dimensóes do suporte social percebido de acordo com o nivel socioeconómico, ser máe de primeira viagem e afeto positivo. Outros estudos com tamanho amostral maior sáo recomendados.

## Introducción

El embarazo constituye un evento vital de alto impacto para la salud de las mujeres^1^. Es considerado como un periodo crítico, marcado por la necesidad de adaptarse a los cambios físicos, psicológicos y sociales que acontecen en cada semana de gestación^2^. Estos ajustes ponen a las madres gestantes en una posición de vulnerabilidad ante incontables estresores, que pueden incidir de forma negativa en el bienestar propio y del feto^3^. Por este motivo, la promoción de la salud mental en esta población ha cobrado fuerza en las últimas décadas y se posiciona como un elemento fundamental para la prestación de servicios de salud perinatal^4^.

Resulta difícil obtener cifras exactas sobre la cantidad de mujeres embarazadas en tiempo real, no obstante, el registro de nacidos vivos permite establecer un estimado de gestaciones llevadas a término en un lugar determinado. Los datos suministrados por el Departamento Administrativo Nacional de Estadística^5^ indican que, para abril de 2021, se reportaron un total de 2.618 nacidos vivos en Santa Marta, Colombia. Sin embargo, son pocas las investigaciones que han abordado los factores psicosociales asociados al bienestar de las madres gestantes en el contexto de la ciudad.

Además de los respectivos controles médicos y seguimientos ginecobstétricos, los cuidados perinatales también apuntan a satisfacer las necesidades de origen social, familiar y personal que tienen relevancia durante el proceso de gestación^6^. En este sentido, el apoyo social surge como variable de interés para la salud perinatal, pues llevar a término el embarazo implica un constante acompañamiento y cuidado por parte de las redes sociales y los vínculos significativos^7^.

El apoyo social se conceptualiza como la información percibida que provoca sentimientos de pertenencia a una red social, donde la persona comparte responsabilidades y de la cual espera recibir afecto y cuidados^8^. Las diferentes fuentes de apoyo que percibe el individuo son vitales para su participación en sociedad y cumplen funciones de acompañamiento a nivel informativo, instrumental, emocional y de valoración positiva^9^.

En este sentido, el apoyo social percibido se refiere a la concepción subjetiva y cognitiva del apoyo social, es decir, a la percepción de disponibilidad para recibir los cuidados, sin que ello precise su materialización a nivel conductual^10^. Implica una valoración subjetiva sobre la calidad del apoyo que la persona recibe de fuentes externas y puede venir de la familia, personas significativas y amigos^11^^,^^12^. La percepción de apoyo social es fundamental durante la gestación y puede llegar a ser más importante para la salud y el bienestar de la madre que recibir apoyo en realidad^9^.

La evidencia indica que el apoyo social percibido se configura como predictor positivo y consistente de la salud perinatal^13^. Este soporte facilita el afrontamiento de los diversos cambios propios de la gestación hasta la subsecuente maternidad^11^. Además, el apoyo social puede comportarse como un factor protector para la lactancia materna exclusiva, en especial en madres primerizas^14^.

Se teoriza que el acompañamiento y los cuidados perinatales brindados por la familia, personas significativas y amigos reduce el impacto de las situaciones estresantes que se experimentan antes, durante y después del parto, promueven el afecto positivo en la mujer y favorecen el apego seguro entre la madre y el hijo a través de la lactancia^15^.

Asimismo, la satisfacción con el apoyo social familiar favorece la autoeficacia, el bienestar subjetivo y la percepción positiva sobre las competencias parentales^16^. La satisfacción con la relación de pareja y el apoyo percibido por personas significativas se relacionan con la calidad de vida y disminuyen el riesgo de presentar síntomas de depresión, ansiedad, estrés generalizado y baja autoeficacia^17^^-^^19^. El apoyo por parte de los amigos, además, puede reducir el estrés parental, los sentimientos de soledad y el riesgo de depresión posparto^20^.

El apoyo percibido por parte de otros actores sociales también influye en el bienestar general de la madre. Por lo general, el cuidado de la mujer durante la gestación está compuesto por un equipo interdisciplinar de enfermeros, médicos, ginecólogos, psicólogos y otros profesionales^21^. Este atendimiento en salud integral se refleja en el apoyo percibido por las gestantes, pues disminuye la incertidumbre, fortalece el nivel de conocimientos relacionados al embarazo y ofrece recursos para tener una experiencia de parto positiva^22^^,^^23^.

En el periodo de gestación ocurren diferentes cambios hormonales y fisiológicos que inciden directamente en las emociones y se intensifican en el tercer trimestre^24^. De este modo, la afectividad, entendida como una característica estable que configura tendencias para experimentar determinados estados de ánimo de forma recurrente o respondiente, se torna relevante para mantener un equilibrio emocional durante el embarazo^25^. El afecto positivo abarca la dimensionalidad placentera de las emociones y se manifiesta a partir de sentimientos de excitación, satisfacción, motivación y orientación al logro^26^.

El afecto positivo favorece la estimulación de emociones placenteras en el embarazo y proporciona a la madre un ambiente de tranquilidad y alegría, que se traduce en mayor bienestar para ella y el feto^22^. En el tercer trimestre, las gestantes dependen en mayor medida de su entorno y requieren atención especial por parte de sus redes sociales; así, el apoyo social promueve estados afectivos positivos y contribuye a la salud mental perinatal^27^.

La literatura indica que el apoyo social percibido es relevante para el bienestar, la afectividad y la calidad de vida de las madres gestantes. Sin embargo, existen muy pocos estudios sobre esta variable en mujeres de la ciudad de Santa Marta. A este respecto, solo se cuenta con el antecedente de Suárez y Gómez^28^, quienes evaluaron 53 adolescentes embarazadas de la comuna seis de la ciudad en el año 2010 y reportaron una asociación negativa entre el apoyo social percibido y la depresión (p<0.018). En el nivel regional, Borda et al.^29^ realizaron un estudio con 151 gestantes de la ciudad de Barranquilla y encontraron niveles altos de apoyo social percibido (89.4%), aunque no evidenciaron asociaciones significativas entre esta variable y los síntomas de depresión.

El presente trabajo realiza un aporte teórico respecto al estudio del apoyo social en la salud gestacional y contribuye a la comprensión de los factores asociados a la salud mental perinatal en el contexto local. Por todo lo anterior, el objetivo de la investigación fue comparar el apoyo social percibido, en las dimensiones de familia, amigos y personas significativas, según variables sociodemográficas, ginecobstétricas y afecto positivo en mujeres gestantes de Santa Marta, Colombia.

## Materiales y Métodos

Se realizó un estudio de enfoque cuantitativo, de diseño comparativo y transversal, llevado a cabo entre marzo de 2020 y febrero de 2021. Participaron 40 mujeres gestantes de la ciudad de Santa Marta, Colombia, beneficiarias de un programa de promoción a la lactancia materna exclusiva implementado por la Universidad del Magdalena. Las mujeres fueron seleccionadas mediante muestreo intencional, en la medida que ingresaban al programa, a partir de los siguientes criterios: a) mayores de 18 años, b) en su tercer trimestre de embarazo y c) residentes en Santa Marta, todos ellos en coherencia con los objetivos y alcance del programa de lactancia materna. Como criterios de exclusión se tuvieron los siguientes: a) presentar algún tipo de discapacidad neurológica o cognoscitiva, b) ser menor de edad, c) tener un periodo de gestación inferior a seis meses y d) residir por fuera de la ciudad de Santa Marta.

Las participantes diligenciaron una ficha de caracterización para recolectar información sociodemográfica respecto a las siguientes variables: edad, estrato socioeconómico, estado civil, nivel de educación, situación laboral. También, para evaluar variables ginecobstétricas: si era el primer embarazo, si la gestación era de alto riesgo y si presentaba algún tipo de restricción de movilidad.

Se aplicó la Escala Multidimensional de Apoyo Social Percibido^11^. Este instrumento se encuentra adaptado en población colombiana y evidenció buenos indicadores de estructura interna, validez convergente y consistencia interna^30^. Se compone de 12 ítems que miden tres dimensiones: apoyo social familiar (ítems 1, 2, 3 y 4), apoyo social por parte de la pareja o personas significativas (ítems 5, 6, 7 y 8) y apoyo social por parte de los amigos (ítems 9, 10, 11 y 12). Cada inciso se evalúa en una escala tipo Likert con las siguientes opciones de respuesta: 1 (casi nunca), 2 (a veces), 3 (con frecuencia) y 4 (siempre o casi siempre). La puntuación total corresponde a la suma de cada ítem respondido, obteniéndose un valor mínimo de 12 y máximo de 48. Puntuaciones altas indican mayor apoyo social percibido por el individuo. En el presente estudio, se encontró un coeficiente alfa de Cronbach aceptable para la escala general (a=0.88) y para las dimensiones de familia (a=0.60), personas significativas (a=0.73) y amigos (a=0.70).

El afecto positivo se midió con la Escala de Afectos Positivos y Negativos^26^. El instrumento está validado en población adulta de Colombia y evidenció una adecuada validez de constructo y consistencia interna^31^. Está conformado por 20 ítems que describen sentimientos y emociones positivas durante el último mes y evalúan dos dimensiones: afecto positivo (ítems 1, 3, 5, 9, 10, 12, 14, 16, 17 y 19) y afecto negativo (ítems 2, 4, 6, 7, 8, 11, 13, 15, 18 y 20). Los reactivos están estructurados en formato Likert y se califican de la siguiente manera: 1 (levemente o casi nada), 2 (un poco), 3 (moderadamente), 4 (bastante) y 5 (extremadamente). Cada dimensión se califica de forma independiente y se obtienen puntuaciones mínimas de 10 y máximas de 50 para cada una. Valores altos indican mayor afecto positivo o negativo. En el presente estudio, solamente se utilizó la subescala de Afecto Positivo, para evitar redundancias en los análisis e interpretación de resultados. Esta subescala de 10 ítems demostró una buena consistencia interna (a=0.82).

Respecto al procedimiento, se ejecutó la recolección de información con los instrumentos de evaluación. Las mujeres gestantes que hicieron parte del estudio pertenecían a un programa de promoción de la lactancia materna exclusiva, llamado “Programa LME”, el cual fue implementado por el equipo de investigadores durante el 2020-2021. En el transcurso del proyecto, se midieron algunas variables asociadas a la salud mental en las madres gestantes, entre ellas el apoyo social percibido y el afecto positivo. La información recolectada fue digitalizada en una base de datos con la herramienta Microsoft Excel y posteriormente almacenada en un repositorio de acceso público^32^. El análisis fue realizado con el software IBM SPSS versión 25.

En relación con el análisis estadístico, se evaluó la consistencia interna de los instrumentos mediante el coeficiente alfa de Cronbach^33^, para el cual se esperan valores superiores a 0,70^34^. Todas las variables fueron dicotomizadas y se calcularon estadísticos descriptivos de frecuencia y porcentaje. En la variable de apoyo social percibido, el punto de corte fue de 8 para cada dimensión (familia, personas significativas y amigos), en un rango entre 4 y 16 puntos posibles. Las variables sociodemográficas, ginecobstétricas y el afecto positivo se tomaron como categóricas, mientras que el apoyo social percibido, en sus tres dimensiones, se mantuvo como variable cuantitativa.

Se aplicó la prueba de Shapiro-Wilk^35^ para explorar la distribución del apoyo social percibido. Los resultados indicaron una libre distribución (p<0.05), por lo cual, para realizar los análisis comparativos, se escogió la prueba no paramétrica U de Mann-Whitney^36^ para dos muestras independientes.

Se aplicó el estadístico de Levene^37^ para comprobar la idoneidad de la U de Mann-Whitney, el cual arrojó varianzas homogéneas entre los grupos (p>0.05), basadas en la mediana y con grados de libertad ajustados, cumpliendo con el criterio de homocedasticidad. No obstante, en muestras pequeñas, la prueba de Mann-Whitney puede arrojar medianas idénticas entre dos grupos y a su vez mostrar diferencias significativas entre ellos^38^.

Cuando los grupos tienen la misma distribución, un cambio en la ubicación mueve las medianas y las medias en la misma cantidad y, por lo tanto, la diferencia en las medianas es la misma que la diferencia en las medias. De esta manera, la prueba U de Mann-Whitney también es una prueba para la diferencia de medias^36^.

Por lo anterior, se escogieron los rangos medios como medida de comparación, pues permiten identificar con mayor exactitud el grupo que obtuvo los rangos más altos en la variable cuantitativa^39^. Se tomaron como significativas aquellas diferencias entre rangos medios que mostraron valores de probabilidad de p<0.05.

Los aspectos éticos de la investigación fueron evaluados y aprobados por el Ministerio de Ciencia, Tecnología e Innovación de Colombia (Minciencias) a través de la Convocatoria 850 de 2019. La participación de las mujeres gestantes en el estudio fue voluntaria; la información recolectada mediante los instrumentos de evaluación se manejó de forma confidencial y anónima, previo consentimiento informado. Para el diseño y ejecución de la investigación, se tuvieron en cuenta los lineamentos éticos y deontológicos para estudios con seres humanos estipulados en la Declaración de Helsinki^40^ y las normas científicas, técnicas y administrativas para la investigación en salud, reglamentadas según la Resolución 8430 de 1993 del Ministerio de Salud y Protección Social colombiano^41^.

## Resultados

Participaron 40 mujeres gestantes de Santa Marta, Colombia, entre 19 y 41 años de edad M=26.48; DE=5.03. El 67% vivía en estrato socioeconómico entre 0 y 2, el 87.5% tenía pareja, el 67.5% había alcanzado el nivel de estudios superiores y el 42.5% trabajaba o realizaba alguna actividad remunerada.

En la Tabla 1 se especifican las frecuencias y porcentajes de las demás variables ginecobstétricas y el afecto positivo en la muestra.

**Tabla 1:** Frecuencias y porcentajes de las variables de estudio (n=40).

Variables	Frecuencia	Porcentaje
Edad		
19 - 28	27	67.5
29 - 41	13	32.5
Estrato		
0 - 2	27	67.5
3 - 4	13	32.5
Tiene pareja	35	87.5
Tiene estudios superiores	27	67.5
Trabaja	17	42.5
Es su primer embarazo	19	47.5
Presenta embarazo con	12	30
riesgo		
Restricciones de movilidad	6	15
Afecto positivo		
Bajo	7	17.5
Alto	33	82.5

Respecto a los análisis comparativos según variables sociodemográficas, se encontró que las mujeres gestantes con estrato socioeconómico medio-alto (3 y 4) percibían mayor apoyo social por parte de sus amigos, en comparación con las mujeres que vivían en estratos bajos (0 a 2) (U=94.5; p=0.01). Las demás variables no mostraron diferencias estadísticamente significativas en las dimensiones del apoyo social percibido (Tabla 2).

**Tabla 2:** Comparación de rangos medios según variables sociodemográficas, ginecobstétricas y afecto positivo (n=40).

Variables			Familia			Personas significativas			Amigos	
	R	U	*Valor* p	R	U	*Valor* p	R	U	*Valor* p	
Edad	19 - 28	20.85	166.0	0.78	22.00	135.0	0.23	21.65	144.5	0.36
	29 - 41	19.77			17.38			18.12		
Estrato	0 - 2	18.17	112.5	0.66	18.85	131.0	0.19	17.50	94.5	0.01*
	3 - 4	25.35			23.92			26.73		
Pareja	Sí	21.39	56.5	0.20	20.00	85.0	0.91	21.53	51.5	0.13
	No	14.30			20.57			13.30		
Estudios superiores	Sí	21.89	138.0	0.27	20.76	169.5	0.83	21.93	137.0	0.26
	No	17.62			19.96			17.54		
Trabaja	Sí	23.85	138.5	0.11	23.06	152.0	0.22	22.35	164.0	0.38
	No	18.02			18.61			19.13		
Primer embarazo	Sí	25.32	108.0	0.01*	24.82	117.5	0.02*	22.32	165.0	0.34
	No	16.14			16.60			18.86		
Embarazo con	Sí	21.17	160.0	0.81	21.38	157.5	0.75	20.71	165.5	0.94
riesgo										
	No	20.21			20.13			20.41		
Restricciones de	Sí	18.50	90.0	0.64	19.00	93.0	0.73	19.75	97.5	0.86
movilidad										
	No	20.85			20.76			20.63		
Afecto positivo	Bajo	12.00	56.0	0.03*	13.21	64.5	0.06	14.00	70.0	0.10
	Alto	22.30			22.05			21.88		

R= rangos medios; U= prueba U de Mann-Whitney; p= nivel de significancia. *Significancia al nivel de 0.05.

En relación con las variables ginecobstétricas, las mujeres que llevaban a término su primer embarazo evidenciaron mayor apoyo social percibido por parte de su familia (U=108.0; p=0.01) y de su pareja u otras personas significativas (U=117.5; p=0.02), en contraste con las mujeres que ya habían tenido un hijo previamente. Las variables de embarazo con riesgo y restricciones de movilidad no mostraron diferencias significativas con el apoyo social.

De igual modo, las mujeres con afecto positivo alto reportaron mayor apoyo social percibido por parte de la familia (U=56.0; p=0.03). Sin embargo, no se encontraron diferencias estadísticas entre los niveles de afectividad y las dimensiones de amigos y otras personas significativas del apoyo social (Tabla 2).

## Discusión

En el presente estudio se realizó un análisis comparativo del apoyo social percibido en mujeres gestantes según variables sociodemográficas, ginecobstétricas y afecto positivo. En primer lugar, se encontraron diferencias significativas en el apoyo social por parte de los amigos según el estrato socioeconómico de las participantes. Si bien existe poca literatura para comparar los resultados, algunas investigaciones permiten orientar el respectivo análisis.

Según lo informado por Bedaso et al.^42^, en un estudio con 493 mujeres gestantes australianas, la dificultad de las madres para acceder y manejar los ingresos económicos incrementaba 3.1 veces las posibilidades de percibir un bajo apoyo social. En esta misma línea Hetherington et al.^43^ encontraron que las mujeres de estrato socioeconómico bajo tenían 3.3 veces más probabilidades de tener un apoyo social deficiente, al evaluar 3.387 gestantes de Canadá.

Tener una capacidad adquisitiva limitada y vivir bajo condiciones socioeconómicas precarias son factores relevantes para el bienestar de las madres gestantes, en tanto que forman la estructura y determinan la extensión de sus redes sociales, las cuales tienden a ser más pequeñas y menos diversas^44^. Por lo anterior, en la medida que el estrato socioeconómico incrementa, también lo hacen los recursos de capital social disponibles, que se hacen evidentes a través los amigos y la comunidad^42^. De esta manera, las mujeres con nivel adquisitivo alto pueden acceder a los servicios de salud mediante sus vínculos personales y redes de apoyo, lo que provoca una mayor disponibilidad de recursos psicosociales generados por la cohesión social^45^.

De otra parte, se encontraron diferencias en la percepción de apoyo familiar entre las madres primerizas y las que habían tenido un embarazo. Estos hallazgos son coherentes con la literatura disponible. Abdollahpour et al.^46^ reportaron una asociación negativa y significativa entre el apoyo familiar y el número de gestaciones en 358 mujeres de Irán, por lo que, mientras más embarazos tenía una madre, menor era el acompañamiento y soporte percibido.

El apoyo de la familia desempeña un rol importante durante el proceso de gestación, en especial cuando ocurre por primera vez. Con la llegada de un nuevo miembro al núcleo familiar, se produce un salto generacional que obliga a reestructurar los roles, funciones y responsabilidades previamente establecidos; este ajuste permite que se puedan brindar los cuidados perinatales pertinentes a la madre y el neonato^47^.

También se encontraron diferencias entre el apoyo social por parte de la pareja (u otras personas significativas) y llevar a término el primer embarazo. A este respecto Azimi et al.^44^ encontraron que el acompañamiento y apoyo de la pareja durante la gestación disminuye el miedo al parto en un 18%, al evaluar un grupo de 270 mujeres embarazadas por primera vez en Irán.

La literatura sugiere que, en la primera gestación, los padres suelen desplegar un portafolio de comportamientos protectores hacia la mujer, que sirven como apoyo durante el proceso y, al mismo tiempo, mitigan la incertidumbre y temores asociados a vivir la experiencia del embarazo primerizo, promueven la percepción de apoyo por parte de la pareja en la madre gestante y fortalecen la relación entre los progenitores antes y después del parto^48^.

Esta experiencia de acompañar activamente la primera gestación genera recursos personales, familiares y sociales en ambas partes, por lo cual, al asumir la llegada de un segundo hijo, se tienen a disposición un conglomerado de conocimientos, redes de apoyo, capital social, estrategias de afrontamiento y habilidades de resiliencia que facilitan el proceso^49^. Lo anterior podría explicar por qué el apoyo social por parte de la pareja fue significativamente diferente en las gestantes primerizas del presente estudio.

Diversos estudios afirman que el apoyo social por parte de la familia y la pareja se relaciona de forma positiva con la salud mental de la madre gestante. Se han reportado menores índices de depresión, ansiedad y estrés generalizado cuando la familia y otras personas significativas ofrecen un apoyo constante a la madre, tanto en el periodo de embarazo como después del parto^50^^,^^51^.

Por otra parte, el presente trabajo se encontraron diferencias entre el afecto positivo y la percepción de apoyo social familiar en las madres gestantes. Aunque la evidencia a este respecto es limitada, Lotero et al.^25^ reportaron menores indicadores de afectividad positiva en el grupo de mujeres que evidenciaba disfunciones familiares, al evaluar una muestra de 229 gestantes de Medellín, Colombia.

Se teoriza que el afecto positivo favorece la construcción de vínculos familiares funcionales y satisfactorios que, consecuentemente, permiten a las mujeres afrontar las experiencias estresantes durante cada trimestre de la gestación y acentúan la percepción de apoyo social familiar^52^. Además, las emociones positivas durante el embarazo guardan relación con un alta de calidad de vida subjetiva, mejor percepción de la salud y mayor satisfacción con las relaciones familiares^53^.

El presente trabajo marca un antecedente teórico importante respecto al estudio del apoyo social percibido en mujeres gestantes de Colombia y establece una línea base para el desarrollo de próximas investigaciones. Los hallazgos constituyen un aporte significativo para la atención de las madres gestantes de Santa Marta, sirven como insumo para formular procesos de intervención en salud mental perinatal y como evidencia para fundamentar acciones afirmativas que busquen promover el bienestar de las madres gestantes, sus familias y su comunidad.

De otra parte, es importante mencionar algunas limitaciones. El estudio se realizó durante la pandemia por COVID-19, por lo cual, los hallazgos deben ser interpretados con prudencia, pues la información recolectada a partir de los instrumentos de evaluación fue tomada durante una contingencia sanitaria sin precedentes y de alto impacto para la salud física y mental de la población general. Además, el tamaño muestral fue reducido, lo que dificulta realizar inferencias estadísticas para todas las mujeres gestantes de la ciudad de Santa Marta.

Por lo anterior, se recomienda implementar nuevas investigaciones sobre el apoyo social percibido y el bienestar de las madres gestantes de la región, con muestras aleatorizadas y representativas. Asimismo, es importante incluir las dimensiones familiares, sociales y de pareja en los servicios de salud perinatal, de manera que los profesionales de la salud brinden una atención integral durante el embarazo.

Por último, resulta conveniente conocer a profundidad el impacto y las consecuencias de la pandemia por COVID-19 en la salud mental perinatal de las mujeres colombianas. Ante ello, es necesario realizar estudios que abarquen los posibles factores psicosociales asociados a nivel local, regional y nacional.

## Conclusiones

Se concluye que las mujeres gestantes de estrato socioeconómico alto presentaron mayor apoyo social percibido por parte de sus amigos. Las madres primerizas mostraron índices más altos de apoyo familiar y apoyo por parte de la pareja u otras personas significativas, en comparación con las mujeres que ya habían experimentado un embarazo. Las gestantes que reportaron alto afecto positivo percibieron mayor apoyo social por parte de sus familias. Se recomienda incorporar las redes sociales de las madres gestantes en los servicios de salud perinatal, que permitan promover el apoyo social durante el embarazo y favorezcan la salud mental.
